# Endogenous Hydrogen Sulfide Is an Important Factor in Maintaining Arterial Oxygen Saturation

**DOI:** 10.3389/fphar.2021.677110

**Published:** 2021-05-31

**Authors:** Yan Huang, Gang Wang, Zhan Zhou, Zhengshan Tang, Ningning Zhang, Xiaoyan Zhu, Xin Ni

**Affiliations:** ^1^ National Clinical Research Center for Geriatric Disorders and Research Center for Molecular Metabolomics, Xiangya Hospital, Central South University, Changsha, China; ^2^ Department of Physiology, Second Military Medical University, Shanghai, China; ^3^ Reproductive medicine center, Department of obstetrics and Gynecology, General Hospital of Southern Theater Command, Guangzhou, China; ^4^ National Clinical Research Center of Kidney Diseases, Jinling Hospital, Nanjing University School of Medicine, Nanjing, China

**Keywords:** hydrogen sulfide, lung, cystathionine-gamma-lyase, SaO2, hypoxia

## Abstract

The gasotransmitter H_2_S is involved in various physiological and pathophysiological processes. The aim of this study was to investigate the physiological functions of H_2_S in the lungs. In the model of mouse with genetic deficiency in a H_2_S natural synthesis enzyme cystathionine-*γ*-lyase (CSE), we found that arterial oxygen saturation (SaO_2_) was decreased compared with wild type mice. Hypoxyprobe test showed that mild hypoxia occurred in the tissues of heart, lungs and kidneys in *Cse* ^-/-^ mice. H_2_S donor GYY4137 treatment increased SaO_2_ and ameliorated hypoxia state in cardiac and renal tissues. Further, we revealed that lung blood perfusion and airway responsiveness were not linked to reduced SaO_2_ level. Lung injury was found in *Cse* ^-/-^ mice as evidenced by alveolar wall thickening, diffuse interstitial edema and leukocyte infiltration in pulmonary tissues. IL-8, IL-1*β*, and TNF-*α* levels were markedly increased and oxidative stress levels were also significantly higher with increased levels of the pro-oxidative biomarker, MDA, decreased levels of the anti-oxidative biomarkers, T-AOC and GSH/GSSG, and reduced superoxide dismutase (SOD) activity in lung tissues of *Cse* ^-/-^ mice compared with those of wild type mice. GYY4137 treatment ameliorated lung injury and suppressed inflammatory state and oxidative stress in lung tissues of *Cse* ^-/-^ mice. A decrease in SaO_2_ was found in normal mice under hypoxia. These mice displayed lung injury as evidenced by alveolar wall thickening, interstitial edema and leukocyte infiltration. Increased levels of inflammatory cytokines and oxidative stress were also found in lung tissues of the mice with hypoxia insult. GYY4137 treatment increased SaO_2_ and ameliorated lung injury, inflammation and oxidative stress. Our data indicate that endogenous H_2_S is an important factor in maintaining normal SaO_2_ by preventing oxidative stress and inflammation in the lungs.

## Introduction

The endogenous gaseous signaling molecule, H_2_S, is mainly generated from l-cysteine through the activity of enzymes, including cystathionine-*γ*-lyase (CSE) and cystathionine-*β*-synthase (CBS) in a broad spectrum of tissues. Endogenous H_2_S is involved in many physiological and pathophysiological processes, such as angiogenesis (Kanagy et al., 2017), glucose homeostasis (Pichette and Gagnon, 2016), neural activity (Tabassum and Jeong, 2019), vascular tone (Sun et al., 2011), and ischemia-reperfusion (I/R) injury in the brain, liver, lungs, kidneys and heart (Wu et al., 2015a). Emerging evidence indicates that H_2_S plays critical roles in inflammatory responses (Sun et al., 2019), oxidative stress (Tabassum and Jeong, 2019), endoplasmic reticulum stress (Wang et al., 2020), and mitochondrial biogenesis (Murphy et al., 2019).

Many studies have shown that H_2_S is benefit to lung injury upon to various insults such as over-ventilation (Ge et al., 2019), hyperoxia (Faller et al., 2013; Li et al., 2013), lipopolysaccharide (LPS) (Zhang et al., 2016), oleic acid-(Wang et al., 2011a), smoke (Sun et al., 2015; Guan et al., 2020), and ischemia/reperfusion (Qi et al., 2014). H_2_S-donating compounds remarkably alleviates acute lung injury (ALI) induced by I/R and LPS by inhibiting the inflammatory responses (Qi et al., 2014; Faller et al., 2018). H_2_S also inhibits production of reactive oxygen species in pulmonary tissues in hyperoxia-induced ALI (HALI) (Faller et al., 2013). However, the physiological functions of H_2_S in the lungs remain largely unknown to date. Madurga et al. (2015) have demonstrated that H_2_S plays a key role in pulmonary vascular development and lung alveolarization in mouse. We questioned whether a deficiency of H_2_S in adult mice leads to dysfunction in lungs. As oxygen saturation in circulation is an important parameter in evaluating pulmonary function, we investigated the arterial blood oxygen saturation (SaO_2_) in *Cse* ^*-/-*^ knockout mice. It was found that SaO_2_ was reduced in these mice, which was reversed by administration of the H_2_S-donating compound GYY4137. Further, we revealed that reduced SaO_2_ was associated with increased levels of inflammation and oxidative stress in the lungs. Moreover, we demonstrated that GYY4137 ameliorated the decreased SaO_2_ induced by 10% hypoxia in wild type (WT) mice and inhibited inflammatory responses and oxidative stress in the lung tissues.

## Materials and Methods

### Animals

Adult male C57BL/6J (8–10week-old) mice were obtained from Shanghai SLAC Laboratory Animal Co. (Shanghai, China). CSE-deficient (*Cse* ^-/-^) mice with a C57BL/6J background were provided by Shanghai Biomodel Organism Co., Ltd. (Shanghai, China) and genotyped as described previously (Tan et al., 2017). All animal procedures were carried out in accordance with the guidelines for the use of laboratory animals published by the People’s Republic of China Ministry of Health (May, 2006) and with the approval of the Ethical Committee of Experimental Animals of Central South University and the Ethical Committee of Experimental Animals of Second Military Medical University. Mice were housed under controlled temperature (22 ± 2°C) and humid (50 ± 10%) conditions with regular light–dark cycles (12h) and were given a standard diet and water *ad libitum*.

Three sets of experiments were performed in the present study. In the first set of experiments, 12 adult male WT and 12 *Cse* ^-/-^ mice (8–10week-old) were randomly divided into two groups, respectively, i. e, control and GYY4137 group (6/group), Mice of GYY4137 group were administered (i.p.) with 50mg/kg GYY4137, while control mice were injected with the same volume of saline. Animals were sacrificed 24h after treatment and blood and tissues were collected. In the second set of experiments, 12 adult male WT and 12 *Cse* ^-/-^ mice were randomly divided into two groups (6/group), respectively. WT and *Cse* ^-/-^ mice were administered (i.p.) 2.8µmol/kg pinacidil or the same volume of saline as the vehicle control. Animals were sacrificed 24h after treatment and blood and tissues were collected. In the third experiment, WT mice were randomly divided into three groups (12/group); two groups of mice were treated with vehicle or GYY4137 (50mg/kg) and immediately subjected to normobaric hypoxia for 24h. The third group was placed under normoxic conditions and treated with vehicle. For hypoxia treatment, mice were placed in a hypoxia box (10% O_2_, 90% N_2_) with a standard diet and water *ad libitum*. GYY4137 and pinacidil were purchased from Sigma-Aldrich (St Louis, MO, United States) and dissolved in saline. The dosages of GYY4137 and pinacidil were based on previous reports (Nagai et al., 1991; Liu et al., 2014).

### SaO_2_ Assessment

Mouse SaO_2_ was noninvasively measured using a MouseOx Plus system (STARR Life Sciences Corp. Oakmont, PA, United States), as described preciously (Sun et al., 2016). Briefly, the mice were anesthetized with pentobarbital sodium (30mg/kg, i.p.). The tail hair was shaved, and a collar clip light sensor was attached to the tail. Then, SaO_2_ was measured using pulse oximetry.

### Tissue Hypoxia Detection

Tissue hypoxia was detected using the Hypoxyprobe-1 Omni Kit (Hypoxyprobe Inc. Burlington, MA, United States) as described previously (Liu et al., 2015; Sun et al., 2016). Briefly, mice were injected (i.p) with pimonidazole (60mg/kg). After 30min, tissues were collected, fixed overnight in 4% PFA, and embedded in paraffin. Paraffin sections (5μm) of the heart, lungs and kidneys were deparaffinized, rehydrated, and endogenous peroxidase was blocked with 0.3% H_2_O_2_. After heat-induced antigen retrieval, the sections were incubated with anti-hypoxyprobe antibody (1:200) overnight at 4°C. The bound antibodies were detected with 1) fluorophore-labeled secondary antibodies (1:1,000, Jackson ImmunoResearch Lab). Nuclei were counterstained with 4′6-diamidino-2-phenylindole (DAPI) (sigma-Aldrich); or 2) the biotin–streptavidin–peroxidase system (UltraSensitive-SP-kit, MaiXin Biotechnology, Fuzhou, China) using diaminobenzidine (Sigma-Aldrich) as the chromogen. Counterstaining was performed with hematoxylin.

### Laser Doppler Blood Flow Analysis

Lung blood perfusion was evaluated using a Laser Doppler image (LDI) analyzer (Moor Instruments, Cambridge, United Kingdom) as described previously (Ono et al., 2002; Shigemura et al., 2005). Before measurement, mice were anesthetized with pentobarbital sodium (30mg/kg, i.p.) and maintained under a rodent respirator. After midline thoracotomy, the chest was opened to expose the lungs. The mice were placed under the instrument, and the lung surface was scanned with a laser. The lung blood perfusion values were recorded and then calculated using the moorFLPI-2 measurement software (Ver. 1.0).

### Lung Functional Assessment

Lung inspiratory resistance (R_i_), expiratory resistance (R_e_), and dynamic compliance (Cdyn) were measured using the AniRes2005 lung function analysis system (SYNOL High-Tech, Beijing China). As described by Ni et al. (Ni et al., 2011) and Wang et al. (Wang et al., 2011b), mice were anesthetized with pentobarbital sodium (30mg/kg, i.p.), tracheotomized, and connected to a mechanical ventilator. The mice were then placed in a whole-body plethysmograph to measure airway pressure and compliance. R_i_, R_e_, and Cdyn were calculated using AniRes 2005 software from the digitized signals of dynamic airway pressure (ΔP) and volume of chamber (ΔV).

### Hematoxylin-Eosin Staining

Mice were sacrificed and lung tissues were collected, fixed overnight in 4% PFA, and embedded in paraffin (4mm). Sections were stained with hematoxylin and eosin (H&E) and scored by two blinded pathologists according to previously described criteria (Yang et al., 2010). Briefly, the criteria were as follows: 0, normal tissue; 1, minimal inflammatory change; 2, no obvious damage to the lung architecture; 3, thickening of the alveolar septae; 4, formation of nodules or areas of pneumonitis that distorted the normal architecture; 5, total obliteration of the field.

### Measurement of H_2_S Production Rate in Pulmonary Tissues

We determined the real-time kinetics of H_2_S production in pulmonary tissues using a miniaturized H_2_S micro-respiration sensor (Model H_2_S-MRCh, Unisense, Aarhus, Denmark) coupled to Unisense PA 2000 amplifier was used as previously described (Zhu et al., 2010). The pulmonary tissues were homogenized, and then homogenate was centrifuged for 5min at 5,000rpm at 4°C to remove any remaining tissue chunks. One ml analysis buffer (1mM L-cysteine and 2mM pyridoxal-5’-phosphate in PBS) at 37°C was added into a temperature-controlled microrespiration chamber (Unisense) inside a well-grounded Faraday cage. To avoid the spontaneous H_2_S oxidation, nitrogen was used to deoxygenate the analysis buffer in the respiratory chamber. After the sensor signals were stabilized, a volume of 50μl pulmonary protein solution (10–20mg) was injected into the chamber, real-time H_2_S production trace was recorded. H_2_S production rates were determined at the initial steepest slopes of each trace. The H_2_S sensor was calibrated after each experiment with freshly prepared anoxic sodium sulfide stock solution (0–100μmol/L) according to the manufacturer's manual, using the same buffer and conditions.

### Immunocytochemistry

Pulmonary tissues were fixed in buffered formalin prior to processing the paraffin sections. Paraffin sections (5µm) were cut, rehydrated and microwaved in citric acid buffer to retrieve antigens. The specific antibodies for CSE were purchased from Santa Cruz Biotechnology (Santa Cruz Biotechnology, Inc. Santa Cruz, CA). Sections were incubated with 3% H_2_O_2_ to inhibit endogenous peroxidases, and then with 10% rabbit serum for 30min to block the unspecific antibody binding. The sections were incubated with CSE antibody (1:200; Santa Cruz Biotech.; Cat# sc-365381) in PBS containing 1% BSA for 24h at 4°C. The bound antibodies were detected with the biotin–streptavidin–peroxidase system (UltraSensitive-SP-kit, MaiXin Biotechnology, Fuzhou, China) using diaminobenzidine (Sigma-Aldrich) as chromogen. Counterstaining was performed with hemalum. Negative controls were performed by substituting primary antibody with a normal serum in same dilution.

### Measurement of IL-1*β*, IL-8, and TNF-*α* Production

The concentrations of IL-1*β*, IL-8, and TNF-*α* were determined using an ELISA kit (Westang Biotech Co., Ltd., Shanghai, China) according to the manufacturer’s instructions.

### Measurement of Reduced and Oxidized Glutathione Content, Catalase, Superoxide Dismutase Activity, Hydrogen Peroxide Concentration, Total Antioxidant Capacity, and Malondialdehyde Levels

Lung tissues (150mg) were homogenized in cold saline and centrifuged at 1,000 × g for 15min and the supernatant was collected. The ratio of reduced/oxidized glutathione (GSH/GSSG), catalase (CAT), superoxide dismutase (SOD) activity, H_2_O_2_ concentration, and total antioxidant capacity (T-AOC) in the lung tissues were measured using a GSH/GSSG colorimetric assay kit, CAT activity assay kit, total SOD activity assay kit, H_2_O_2_ detection kit, and T-AOC detection kit, respectively. All kits were purchased from Westang Biotech Co., Ltd.

For measurement of malondialdehyde (MDA) levels, 100°mg of lung tissue was homogenized in 1ml of 1.15% KCl solution containing 0.85% NaCl. Homogenates were then centrifuged at 1,500 × g for 15min, and the supernatant was collected. The levels of MDA were determined using a MDA assay (Westang Biotech Co., Ltd.).

### Isolation of Mitochondria

Isolation of mitochondria was performed using the Mitochondria Fractionation Kit (Beyotime, China). Briefly, mouse lung tissues were quickly removed and placed in beakers containing chilled (4°C) isolation media (0.25M sucrose, 10mM Tris–HCl buffer, pH 7.4, 1mM EDTA and 250μg BSA/ml. The tissues were minced and washed three times with the isolation media to remove adhering blood and 10% (w/v) homogenates were prepared using homogenizer. The nuclei and cell debris were sedimented by centrifugation at 600g for 10min and discarded. The supernatant was subjected to a further centrifugation at 10000g for 10min. Mitochondrial pellets were suspended in the isolation medium. Respiratory control ratio (RCR) was used to assess the quality of isolated mitochondria.

### Measurement of Mitochondrial Superoxide Production, Membrane Potential and ATP Concentration

Mitochondrial superoxide was measured by using a MitoSOX™ Red mitochondrial superoxide indicator (Invitrogen, United States) as described previously (Wang et al., 2014). Briefly, the 5mM stock solution was prepared by adding 13μl DMSO to each tube of MitoSOX™, and then the stock solution was diluted with HBSS to a 5μM working solution. Next, 190μl MitoSOX™ working solution was added to each well of a 96-well cell culture plate, then 10μl of fresh prepared mitochondrial was added and incubated for 10min at 37°C in dark. The plates were measured by the microplate reader with the excitation light of 510nm and emission light of 580nm.

JC-1 probe (Beyotime, China) was employed to measure mitochondrial depolarization. Briefly, 180μl of diluted JC-1 working solution was added to each well of a 96-well cell plate, and then 20μl of mitochondrial solution was added. Mitochondrial membrane potentials were monitored by determining the relative amounts of dual emissions from mitochondrial JC-1 monomers and polymers. The fluorescence value was detected by a microplate reader: for JC-1 monomer with the excitation light of 490nm and emission light of 530nm; for JC-1 polymer with the excitation light of 525nm and emission light of 590nm. The ratio of fluorescence at 590 vs. 530nm emission was used for measuring the mitochondrial membrane potential.

The ATP concentrations were measured with enhanced ATP assay kit (Beyotime, China) according to the manufacture’s protocol. Briefly, 100µl of ATP working solution was added to 1.5ml EP tubes and incubated for 5min at room temperature. Next 10µl fresh prepared mitochondrial solution was transferred to the ATP working solution. And the amount of luminescence emitted was measured with a luminometer (Promega, Madison, WI, United States) immediately. The luminescence data were normalized against sample protein amounts.

### Western Blot Analysis

Tissue samples were lyzed with cold RIPA lysis buffer (Beyotime, China) and followed by centrifuging at 12,000 × g for 15min at 4°C. The supernatants were then collected. The protein concentration was determined by Pierce™ BCA Protein Assay Kit (Thermo Fisher Scientific, MA, United States). Then, tissue extracts were mixed with 4× loading buffer containing 250mmol/L Tris-HCl, 10%SDS, 0.5% bromophenol blue, 50% glycerol and 7.5% DTT at pH 6.8. Samples were heated to 99°C for 10min before loading on a gel. The samples were separated by 10% SDS-PAGE and subsequently transferred to nitrocellulose membranes (Millipore Corp, Bedford, MA). The membranes were incubated with blocking buffer (Tris-buffered saline containing 0.1% Tween-20 and 5% skimmed milk powder) for 2h at room temperature, and then incubated with primary antibodies against CSE (Santa Cruz Biotech.; Cat# sc-365381), CBS (Santa Cruz Biotech.; Cat# sc-133208) or *β*-actin (Abcam; Cat# ab-8226) at 4°C overnight. After incubation with a secondary horseradish peroxidase-conjugated IgG (Santa Cruz) for 1h at room temperature, immunoblots were visualized using the enhanced chemiluminescence Western blotting detection system (Millipore). The chemiluminiscent signal from the membranes was quantified by a GeneGnome HR scanner using GeneTools software (SynGene).

### Statistical Analysis

Statistical analyses were performed using SPSS Ver. 20. All data are expressed as mean ± standard error of the means (SEM) and normal distribution was assessed using the Shapiro–Wilk test. Statistical significance was determined according to sample distribution and homogeneity of variance. Statistical comparisons between two groups were determined by two-tailed Student’s *t*-test. One-way analysis of variation (ANOVA) using Bonferroni’s post hoc test and the Kruskal–Wallis test with Dunn’s post hoc test was performed for multi-group analysis. *p* < 0.05 was considered statistically significant.

## Results

### *Cse^-/-^* Mice Display Reduced SaO_2_ and H_2_S Donor Treatment Increases SaO_2_ Level

**FIGURE 1:**
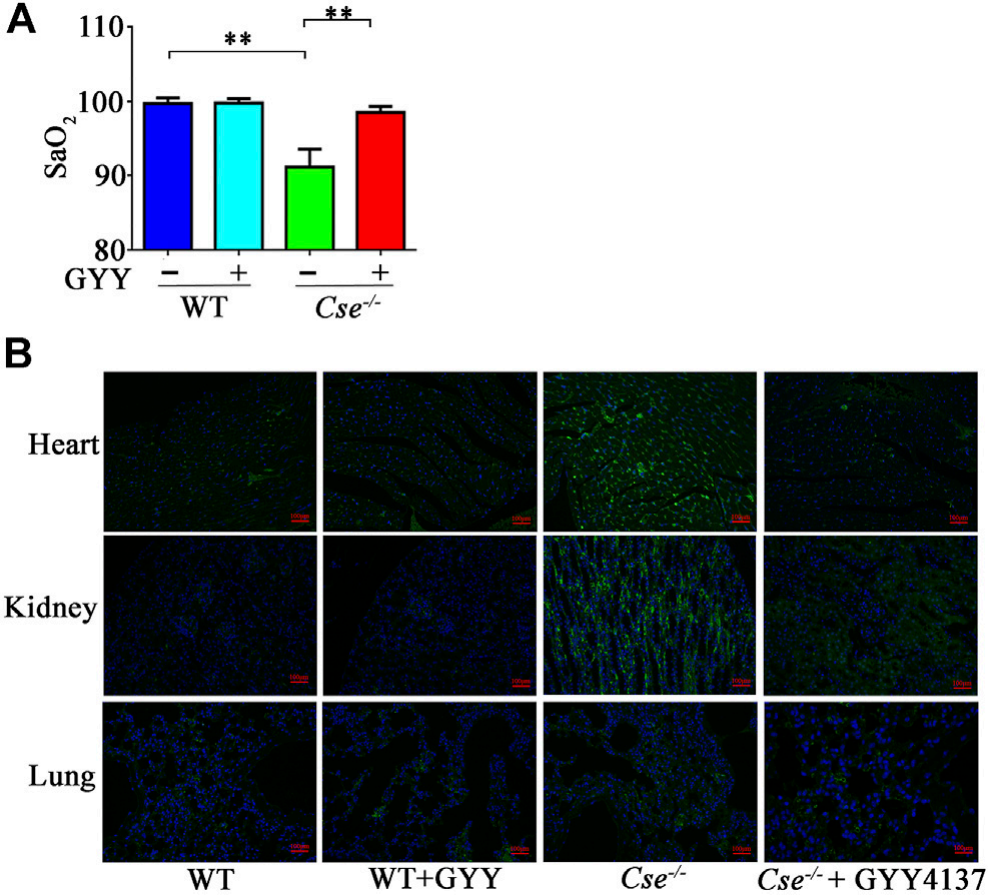
Reduced H_2_S levels contribute to decreased SaO_2_ and tissue hypoxia in *Cse* ^*-/-*^ mice. WT and *Cse* ^*-/-*^ mice were administered with saline or GYY4137 (50mg/kg). Twenty-four hours after injection, the mice were anesthetized and SaO_2_ level was measured. Some mice were then injected (i.p) with Hypoxyprobe solution. The animals were sacrificed at 30min after injection of Hypoxyprobe solution. Biopsies of kidneys, lungs and heart were fixed. Hypoxyprobe signals were analysis by using fluorescent analysis. **(A),** SaO_2_ level in WT and *Cse* ^*-/-*^ mice with saline or GYY4137 treatment. **(B),** representative images of Hypoxyprobe signals in the heart, lungs, and kidneys in WT and *Cse* ^*-/-*^ mice with saline or GYY4137 administration. Data were expressed as mean ± SEM; *n* = 8–12 per group; **p < 0.05.

We previously demonstrated that H_2_S plays an important role in oxygen transport (Wang et al., 2021). In the present study, we found that SaO_2_ was notably decreased in *Cse* ^-/-^ mice compared with that in WT mice (Figure 1A). We next assessed tissue hypoxia in the kidney, heart and lung using the Hypoxyprobe method. Compared with WT mice, immunofluorescence analysis of Hypoxyprobe signals showed slightly elevated staining in cardiac and renal but not in pulmonary tissues of *Cse* ^-/-^ mice (Figure 1B). *Cse* ^*-/-*^ mice showed a remarkably reduced H_2_S level, as demonstrated by our and other studies (Yang et al., 2008; Liu et al., 2014; Wang et al., 2021). We then investigated whether decreased SaO_2_ and tissue hypoxia are attributed to H_2_S deficiency in *Cse* ^-/-^ mice. As shown in Figures 1A,B, treatment of *Cse* ^-/-^ mice with a slow-releasing H_2_S donor, GYY4137 led to an increase in SaO_2_ and a decrease in hypoxyprobe signals in heart and kidney of *Cse* ^-/-^ mice.

### Lung Airway Resistance and Blood Flow Are Not Associated With SaO_2_ Level in *Cse* ^*-/-*^ Mice

**FIGURE 2:**
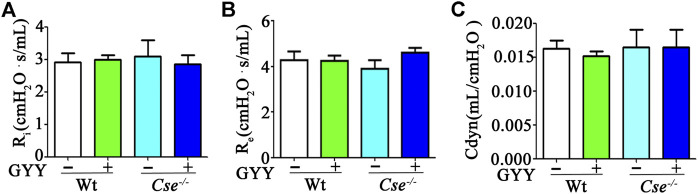
H_2_S deficiency does not affect lung airway resistance. WT and *Cse* ^-/-^ mice were administered with saline or GYY4137 (50mg/kg). Twenty-four hours later, the mice were anesthetized and then lung inspiratory resistance (R_i_) **(A)**, expiratory resistance (R_e_) **(B)**, and dynamic compliance (Cdyn) **(C)** were analyzed by using AniRes2005 lung function analysis system. The data were expressed as Mean ± SEM; *n* = 8–11 per group.

Given that airway resistance and lung blood perfusion can affect SaO_2_ level, we examined these parameters. Airway responsiveness was evaluated using pulmonary ventilation. As shown in Figures 2A–C, there were no differences in R_i_, R_e_, and Cdyn between WT and *Cse* ^-/-^ mice, and GYY4137 treatment had no effect on airway responsiveness.

**FIGURE 3:**
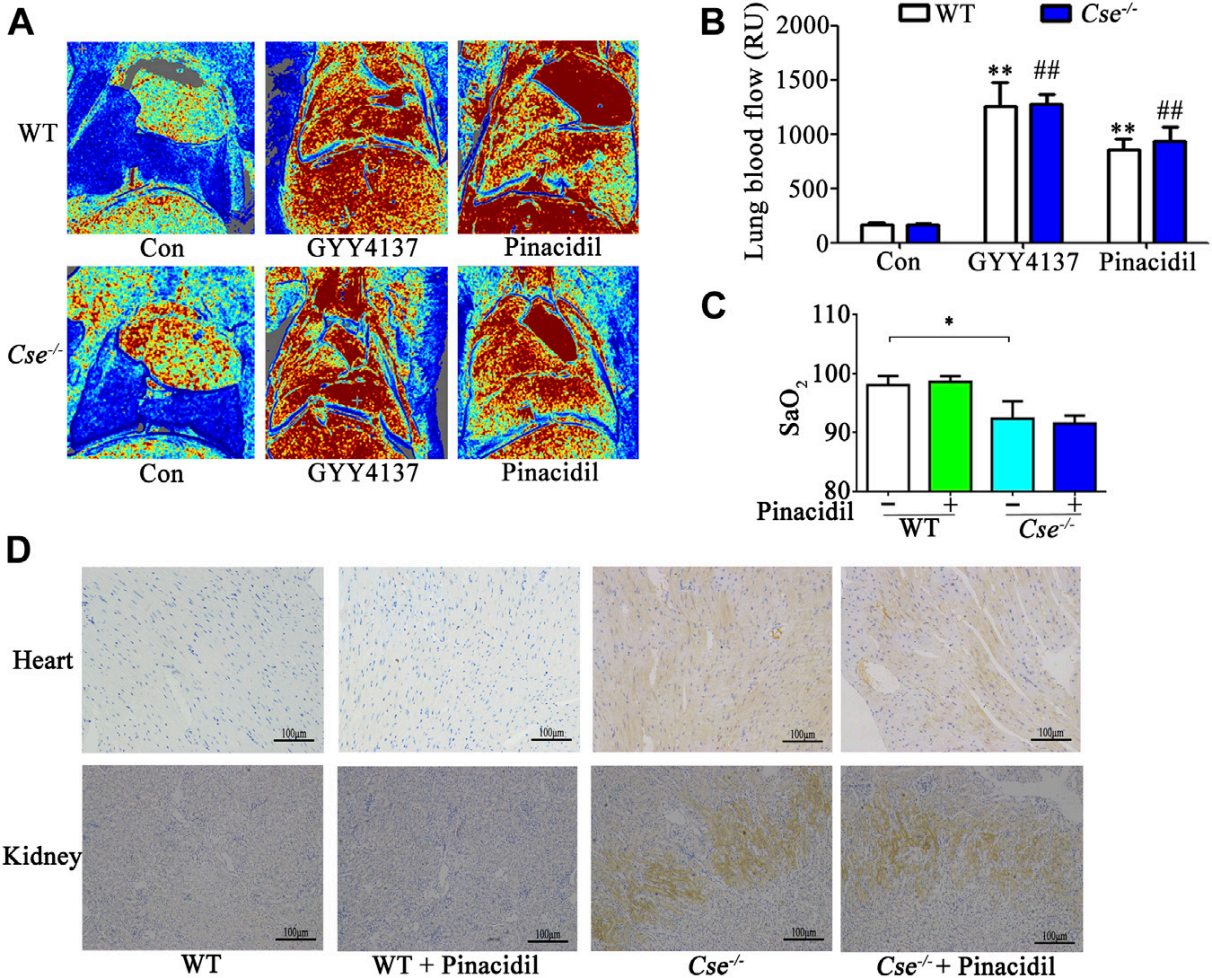
Increased lung blood flow does not affect SaO_2_ and tissue hypoxia in kidney and heart. WT and *Cse* ^-/-^ mice were administered with saline, pinacidil (2.8μmol/kg), or GYY4137 (50mg/kg). Twenty-four hours later, the mice were anesthetized and then lung blood flow and SaO_2_ was evaluated. Some mice were injected with Hypoxyprobe solution, then the mice were sacrificed at 30min after injection. Biopsies of kidneys and heart were fixed. Hypoxyprobe signals were detected by using immunocytochemistry analysis. (**A)**, representative LDI analysis of lung blood flow. **(B)**, cumulative data of lung blood flow in the mice. Data were expressed as Mean ± SEM; *n* = 6–10 per group; **p < 0.01 vs. WT, ^##^ *p* < 0.01 vs. *Cse* ^-/-^. **(C),** SaO_2_ level in WT and *Cse* ^-/-^ mice with saline or pinacidil treatment. Data were expressed as Mean ± SEM; *n* = 8–9 per group; *p < 0.05. **(D),** representative images of Hypoxyprobe signals in the heart and kidneys in WT and *Cse* ^-/-^ mice with saline or pinacidil treatment.

H_2_S is an important vasodilator by controlling K_ATP_ channel activity. *Cse* ^-/-^ mice therefore display hypertension because increased contractility of arterials (Yang et al., 2008). H_2_S can increase lung blood perfusion by relaxing blood vessels in lungs. Thus, pinacidil, a K_ATP_ channel activator, was used to determine whether reduced SaO_2_ and tissue hypoxia in *Cse* ^-/-^ mice are linked to hypertension. In a previous study, we demonstrated that H_2_S and pinacidil could both reduce the blood pressure in *Cse* ^-/-^ mice (Wang et al., 2021). Here, H_2_S and pinacidil treatment increased lung blood perfusion in WT and *Cse* ^-/-^ mice (Figures 3A,B). However, pinacidil treatment had no effect on SaO_2_ and hypoxia in heart and kidney (Figures 3C,D), indicating that H_2_S maintenance of SaO_2_ is not associated with lung blood perfusion.

### *Cse* ^*-/-*^ Mice Display Pathological Alternation and Inflammation in Pulmonary Tissues and GYY4137 Treatment Ameliorates Pathological Alternations and Inflammation in the Lungs

**FIGURE 4:**
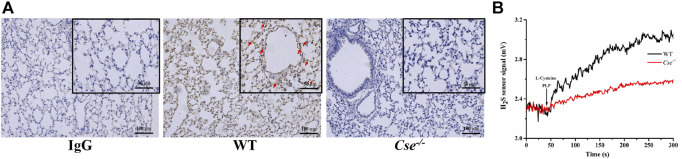
CSE expression localization and H_2_S production in the lung tissue of WT and *Cse^-/-^* mice. **(A)**, CSE expression in lung tissues of WT and *Cse^-/-^* mice was analyzed by immunocytochemistry. Representative images of CSE expression in the lung tissue of WT and *Cse^-/-^* mice. Arrows indicate CSE positive staining. **(B),** H_2_S production rate in the lung tissues of WT and *Cse^-/-^* mice. Black trace, representative trace of H_2_S production in the homogenate of lung tissue in WT mice. Red trace, representative trace of H_2_S production in the homogenate of lung tissue in *Cse^-/-^* mice.

Gas exchange occurs at the alveolocapillary barrier. We hypothesized that CSE deficiency causes histological alternation in the lungs, thereby leading to reduction of SaO_2_. Since H_2_S produced locally in the lungs might be critical for maintenance of normal morphology, we firstly examined CSE expression distribution and capacity of H_2_S production in WT mice and *Cse* ^*-/-*^ mice. As shown in Figure 4A, CSE positive staining was identified in alveolar epithelial cells, vascular endothelial cells, and smooth muscle cells in WT mice. There was no obvious staining of CSE in the lungs of *Cse* ^*-/-*^ mice. The lung tissues were able to produce H_2_S upon to L-cysteine supply. H_2_S production rate was remarkably reduced in the lung tissues of *Cse* ^*-/-*^ mice (Figure 4B).

**FIGURE 5:**
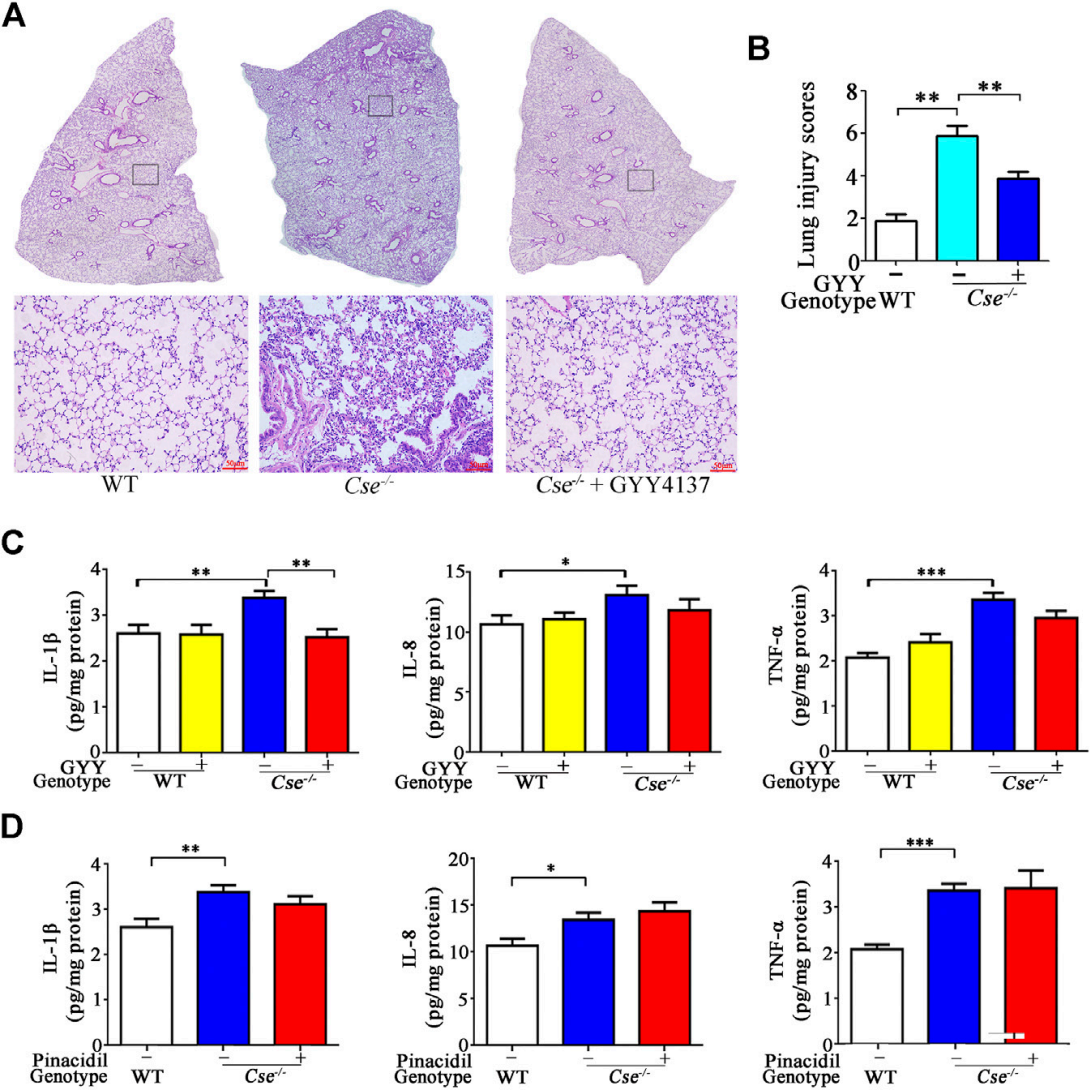
GYY4137 treatment ameliorates pathological alternation and inhibits inflammation in lung tissues in *Cse^-/-^* mice. WT and *Cse* ^-/-^ mice were administered with saline, GYY4137 (50mg/kg) or pinacidil (2.8μmol/kg). Twenty-four hours after injection, the mice were sacrificed, and the lung tissues were then collected for morphological analysis and determination of proinflammatory cytokines. **(A)**, representative images of H&E staining of lung tissues of WT and *Cse* ^-/-^ mice with saline or GYY4137 treatment. **(B)**, cumulative data of lung injury scores of WT and *Cse* ^-/-^ mice with saline or GYY4137 treatment. Data were expressed as mean ± SEM; *n* = 8 per group. **(C)**, IL-8, IL-1*β* and TNF-*α* levels in lung tissues of WT and *Cse* ^-/-^ mice with saline or GYY4137 treatment. Data were expressed as Mean ± SEM; *n* = 8 per group. **(D)**, IL-8, IL-1*β*, and TNF-*α* levels in lung tissues of WT and *Cse* ^-/-^ mice with saline or pinacidil treatment. Data are expressed as mean ± SEM; *n* = 8–11 per group; *p < 0.05, **p < 0.01, ***p < 0.001.

Histological analysis showed that alveolar was thickened, alveolar air space was decreased, and diffuse interstitial edema and leukocyte infiltration were found in *Cse* ^*-/-*^ mice compared with WT mice (Figure 5A). Assessment of histopathologic score showed that *Cse* ^-/-^ mice obtained higher histopathologic score in pulmonary tissues than WT mice (Figure 5B). GYY4137 treatment ameliorated alveolar thickening, diffuse interstitial edema, and leukocyte infiltration, and decreased histopathologic score in *Cse* ^*-/-*^ mice.

As histological analysis showed inflammation in the lungs of *Cse* ^-/-^ mice, we therefore determined inflammatory cytokines in the lungs of *Cse* ^-/-^ mice. As shown in Figure 5C, the pro-inflammatory cytokine IL-8, IL-1*β*, and TNF-*α* levels were elevated in lung tissues of *Cse* ^-/-^ mice compared with that of WT mice. Administration of GYY4137 reversed the levels of these pro-inflammatory cytokines. However, pinacidil treatment had no effect on IL-8, IL-1*β*, or TNF-*α* levels (Figure 5D).

### Oxidative Stress Occurs in the Lungs of *Cse* ^*-/-*^ Mice and GYY4137 Treatment Suppresses Oxidative Stress in Pulmonary Tissues

**FIGURE 6:**
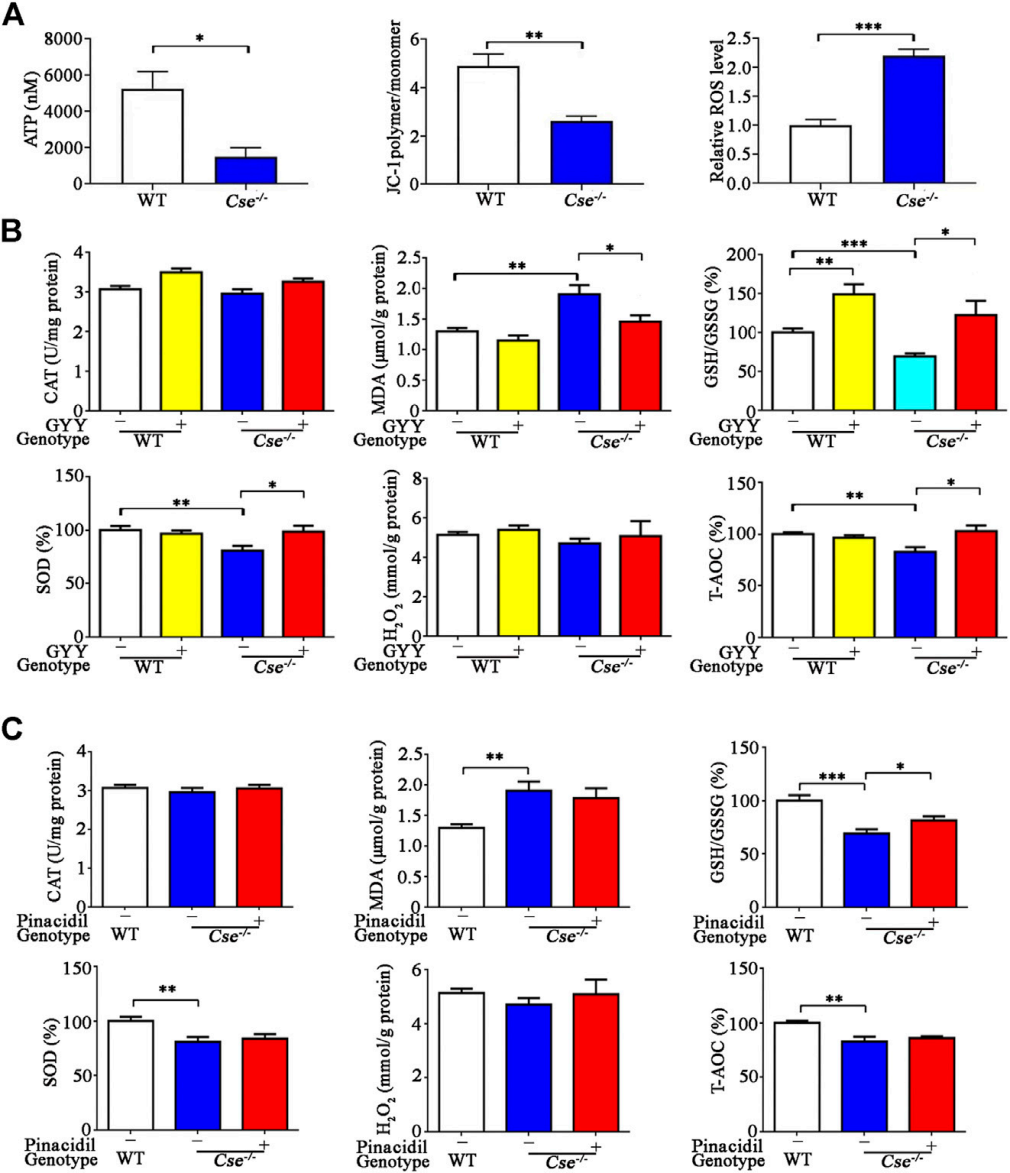
*Cse^-/-^* mice exhibit mitochondria dysfunction and oxidative stress in lung tissues and GYY4137 treatment ameliorated oxidative stress in lung tissues. **(A)**, mitochondrial function in the lungs of WT and *Cse* ^*-/-*^ mice. Mitochondria were isolated from lung tissues. ATP production, membrane potential and ROS production were determined as described in Materials and Methods. **(B)**, parameters of oxidative status in lung tissues of WT and *Cse* ^-/-^ mice with GYY4137 treatment. WT and *Cse* ^−/-^ mice were administered with saline or GYY4137 (50mg/kg). Mice were sacrificed at 24h after injection. The lung tissues were collected for determination of GSH/GSSG, CAT, SOD, H_2_O_2_ and T-AOC. **(C)**, parameters of oxidative status in lung tissues of WT and *Cse* ^-/-^ mice with pinacidil treatment. WT and *Cse* ^-/-^ mice were administered with saline or pinacidil (2.8μmol//kg). After 24h, the mice were sacrificed at 24h after injection. The lung tissues were collected for determination of GSH/GSSG, CAT, SOD, H_2_O_2,_ and T-AOC. Data are expressed as mean ± SEM; *n* = 8–11 per group; *p < 0.05, **p < 0.01, ***p < 0.001.

H_2_S deficiency is associated with oxidative stress in various tissues (Yang et al., 2015; Bai et al., 2018; Liu et al., 2020). Our previous study had shown that CSE deficiency leads to mitochondrial dysfunction in adrenal gland and subsequently results in oxidative stress (Wang et al., 2014, Wang et al., 2018). We therefore examined mitochondrial function in the lungs of *Cse* ^*-/-*^ mice. As shown in Figure 6A, mitochondrial ATP production and membrane potential were significantly reduced whilst ROS level was significantly increased in the pulmonary tissues of *Cse* ^*-/-*^ mice compared with those of WT mice.

We then determined the oxidative status in pulmonary tissues by measuring the pro-oxidative biomarker MDA and several anti-oxidative biomarkers including H_2_O_2_, GSH/GSSG, and T-AOC, as well as the antioxidant enzymes SOD and CAT. As shown in Figure 6B, oxidative status was remarkably elevated in lung tissues of *Cse* ^-/-^ mice compared with those of WT mice as evidenced by an increase in MDA and a decrease in GSH/GSSG, T-AOC, and SOD activity. GYY4137 treatment reduced MDA and increased GSH/GSSG, T-AOC, and SOD level in lung tissues of *Cse* ^-/-^ mice. As blood perfusion in the lung might be associated with oxidative stress, we examined whether pinacidil affected these responses. As shown in Figure 6C, pinacidil treatment did not affect the levels of MDA, T-AOC, and SOD, but increased the GSH/GSSG level in *Cse* ^-/-^ mice.

### H_2_S Treatment Elevates SaO_2_ and Attenuates Histological Alternation, Inflammation, Oxidative Stress in Pulmonary Tissues in the Mice With Hypoxia Insult

**FIGURE 7:**
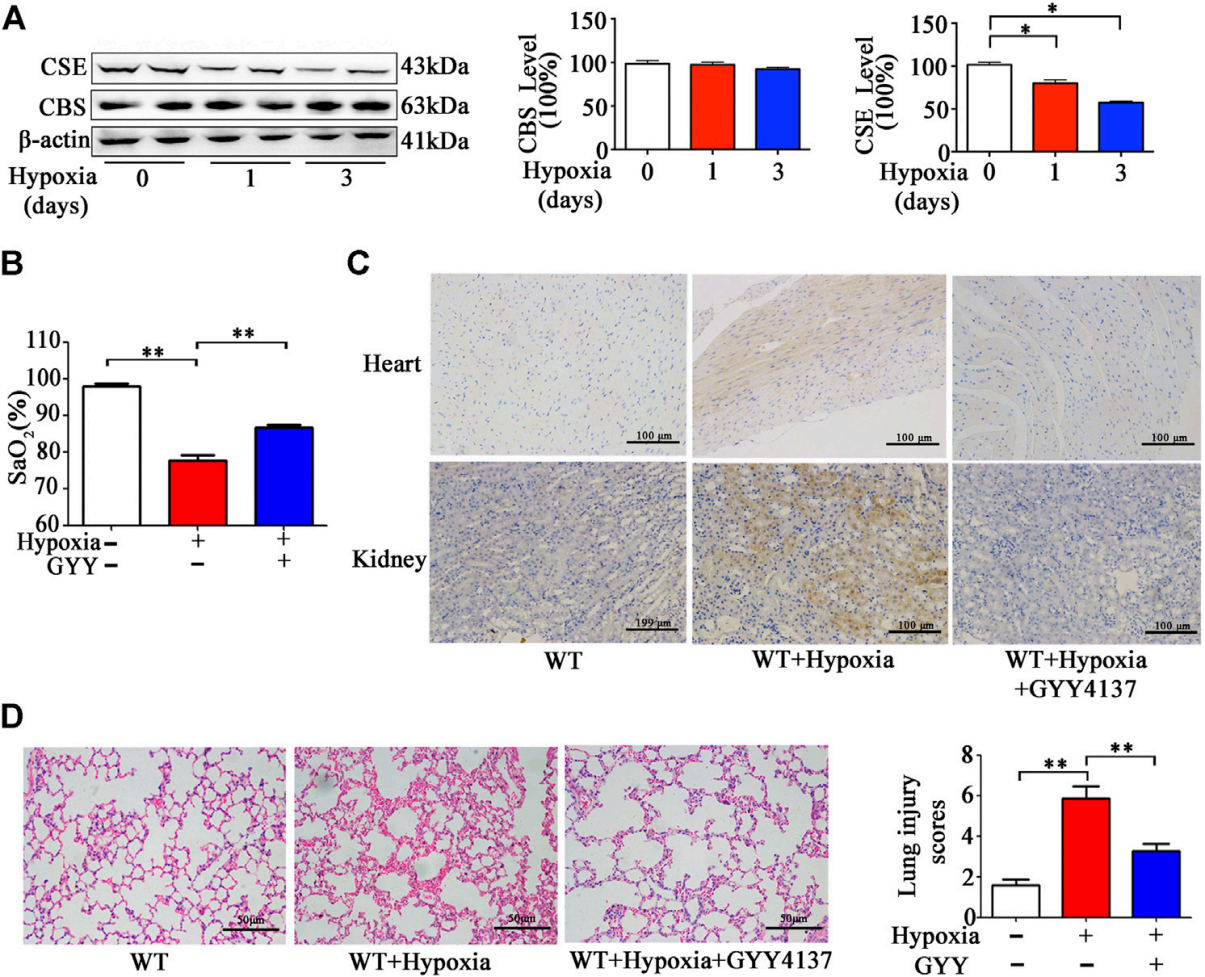
GYY4137 treatment increases SaO_2_ level and attenuates pathological alternation in the lungs and Hypoxyprobe signals in heart and kidneys of the mice with hypoxia insult. **(A)**, CBS and CSE levels in lung tissues of the mice with hypoxia insult. Mice were exposed to normobaric hypoxia (10% oxygen) for the indicated times. Levels of CSE and CBS in lung tissues were determined by western blotting analysis. Representative protein bands are shown to the left of the cumulative histograms. (**B–D)**, the effects of GYY4137 on SaO_2_ and lung morphology in the mice with hypoxia insult. Mice were treated with GYY4137 (50mg/kg) and immediately subjected to normobaric hypoxia for 24h. Then, the mice were anesthetized for determination of SaO_2_ level. **(B)**, Some mice were sacrificed at 30min after injection of Hypoxyprobe solution. Biopsies of kidneys, and heart were fixed for Hypoxyprobe signals detection by using immunocytochemistry. **(C)**, panel: representative images of Hypoxyprobe signals in the hearts and kidneys. The lung tissues were collected and fixed for H&E staining and lung injury scores were then calculated. **(D)**, Data are expressed as mean ± SEM; *n* = 8–11 per group; *p < 0.05, **p < 0.01.

Prior studies have demonstrated that H_2_S production in tissues and H_2_S levels in circulation were significantly reduced in response to hypoxia (Wu et al., 2015b). We hypothesized that decreased H_2_S production in the lungs could aggravate tissue damage and hypoxia. At first, we examined the CSE and CBS expression levels in pulmonary in the mice exposed to 10% O_2_ for 1–3days. As shown in Figure 7A, CSE, but not CBS expression was remarkably reduced in the lung tissues after hypoxia for 1 and 3days, suggesting that H_2_S production is reduced in the lungs upon to hypoxia insult.

Then we investigated the effects of H_2_S donor treatment on SaO_2_, histological injury, inflammation and oxidative status in pulmonary tissues of the mice upon to hypoxia insult. As expected, SaO_2_ level was decreased in the mice under hypoxia (10% O_2_) for 24h (Figure 7B). SaO_2_ level was higher in the mice with GYY4137 treatment compared those with vehicle treatment. Hypoxyprobe signals in lungs, heart and kidneys were ameliorated by GYY4137 treatment (Figure 7C). Histological analysis showed that leukocyte infiltration and interstitial edema occurred, and alveolar space was diminished in pulmonary tissues in the mice exposed to 10% O_2_ for 24h. GYY4137 treatment significantly attenuated these pathological changes and reduced the lung pathological injury scores (Figure 7D).

**FIGURE 8:**
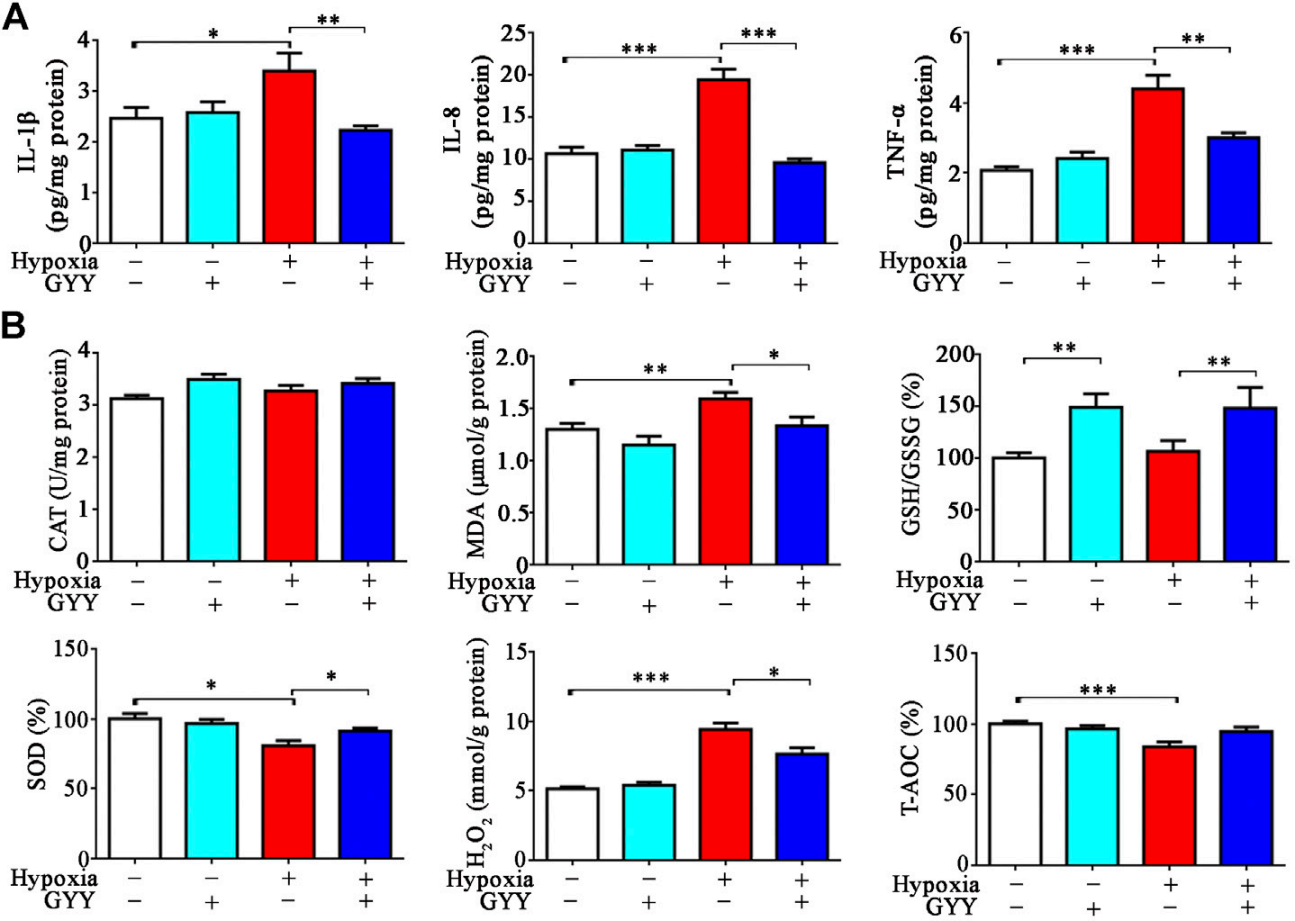
GYY4137 treatment reduces the levels of proinflammatory cytokines and improves oxidative stress in lung tissues of the mice with hypoxia insult. H_2_S alleviates hypoxia-induced inflammation and oxidative stress in the lungs. Mice were treated with GYY4137 (50mg/kg) and immediately subjected to normobaric hypoxia for 24h. Mice were then sacrificed, and lungs tissues were collected for determination of proinflammatory cytokine levels and oxidative stress parameters. **(A)**, IL-8, IL-1*β*, and TNF-*α* levels in lung tissues. **(B)**, GSH/GSSG, CAT, SOD, H_2_O_2_ and T-AOC in lung tissues. Data are expressed as mean ± SEM; *n* = 8–11 per group; *p < 0.05, **p < 0.01, ***p < 0.001.

In consistence with prior studies (Li et al., 2020), inflammatory cytokines IL-1β, IL-8, and TNF-α levels were significantly increased in lung tissues of the mice 10% O_2_ for 24h. GYY4137 treatment decreased the levels of these proinflammatory cytokines (Figure 8A). As expected, oxidative stress occurred in lung tissues in the mice under hypoxia. The pro-oxidative biomarker MDA was increased and the anti-oxidative biomarkers H_2_O_2_ and SOD were significantly decreased in the mice exposed to hypoxia (Figure 8B). GYY4137 administration reversed the above effects. However, GSH/GSSG level was not significantly changed in hypoxia group compared with normoxia group. GYY4137 administration increased GSH/GSSG level.

## Discussion

In this study, we have demonstrated that H_2_S is an important factor in maintenance of SaO_2_ under physiological condition. These effects are associated with its protection of the lungs against oxidative stress and inflammatory responses.

SaO_2_, the ratio of oxygenated hemoglobin (HbO_2_) and total hemoglobin, in the range of 96–100% is considered normal. In this study, we found that SaO_2_ in *Cse* ^-/-^ mice was around 90.48%, indicating that the mice were underwent mild hypoxia. Hypoxyprobe test confirmed that hypoxia occurred in tissues such as heart and kidney in *Cse* ^-/-^ mice. Abnormal SaO_2_ is associated with many pathophysiological processes, in particular, pulmonary disorders, such as abnormal lung blood perfusion, impaired pulmonary ventilation function and acute and chronic lung injury. In humans, reduced SaO_2_ is likely to be due to smoking or pollution-related chronic obstructive airway pulmonary disease (COPD) or early interstitial lung disease and pulmonary fibrosis. Congenital heart disease can change the pathway of blood through the heart and decrease blood perfusion in the lungs to reduce gas exchange (Rhodes et al., 2008). In the present study, we revealed that lung airway resistance was not changed in *Cse* ^-/-^ mice, and increased blood perfusion did not affect SaO_2_ level in *Cse* ^-/-^ mice. However, lung injury including thickened alveolar wall, diffuse interstitial edema and leukocyte infiltration occurred in *Cse* ^-/-^ mice, which suggests that histological alternation in the lungs contributes to reduced SaO_2_ level in these mice.

The dysregulation of redox homeostasis and chronic inflammatory processes represent interdependent factors implicated in the pathogenesis of multiple diseases, including cardiovascular diseases, neurodegenerative diseases, chronic lung disease, and diabetes (Biswas, 2016). Both oxidative stress and inflammation are orchestrated to accentuate each other and induce progressive damage. Currently, H_2_S generation has been reported to be involved in a variety of acute and chronic inflammatory lung diseases, such as ALI (Ali et al., 2018) and COPD (Sun et al., 2015). The protective role of H_2_S in lung disease is associated with anti-inflammatory and antioxidant activities. H_2_S treatment can alleviate lung injury induced by LPS (Zhang et al., 2016; Ali et al., 2018), ventilation (Ge et al., 2019) and smoking (Sun et al., 2015) by regulating inflammation and oxidative stress. Our previous study has demonstrated that circulatory H_2_S level is decreased in *Cse* ^-/-^ mice (Wang et al., 2021), which has been supported elsewhere (Liu et al., 2014; Wang et al., 2021). In the present study, we revealed that pathological changes in the lung tissues including interstitial edema, leukocyte infiltration, inflammation, and redox imbalance were reversed by administration of GYY4137 in *Cse* ^-/-^ mice. These data suggest that H_2_S is an important factor to suppress inflammation and oxidative stress in the lungs, thereby maintaining physiological homeostasis of the lungs.

CSE utilizes L-cysteine as a substrate to generate H_2_S. Bibli et al. (Bibli et al., 2019) demonstrated that CSE deficiency in endothelial cells was associated with endothelial dysfunction, which was reversed by treatment with H_2_S donor. Zhang et al.(2013) showed that deletion of CSE promotes ovalbumin-induced airway hyper-responsiveness and aggravates airway inflammation, which is alleviated by treatment with the H_2_S donor NaHS. In a cell model, CSE knockdown and administration of the CSE inhibitor PAG significantly enhances ox-LDL-induced TNF-*α* generation, which is inhibited by exogenous H_2_S (Wang et al., 2013). Our study showed that CSE was mainly expressed alveolar epithelial cells and vascular endothelial cells in the lungs. As mentioned, H_2_S donor could inhibit inflammation, and oxidative stress in the lungs of *Cse* ^-/-^ mice. Taken together, it might suggest that H_2_S contributes to the physiological functions of CSE in the lungs.

It is known that mitochondria dysfunction can lead to oxidative stress and vice versa. Our previous studies have shown that CSE and CBS dysregulation and reduced H_2_S generation result in mitochondria dysfunction and subsequently lead to oxidative stress in adrenal glands in mice (Wang et al., 2014; 2018). Some studies also demonstrated that H_2_S metabolism contributes to maintenance of mitochondria function in various tissues (Guo et al., 2012; Módis et al., 2016; Meng et al., 2018; Murphy et al., 2019). Consistently, it was found that mitochondria dysfunction occurred in the pulmonary tissues in *Cse* ^-/-^ mice as evidenced by reduced membrane potential and ATP production and increased ROS level. Thus, mitochondria injury and oxidative stress aggravate lung injury in *Cse* ^-/-^ mice.

We previously demonstrated that circulatory H_2_S levels are significantly decreased in the mice exposure to hypoxia (Wang et al., 2021). Here, we showed that CSE but not CBS level was reduced in pulmonary tissues under hypoxia, and H_2_S donor ameliorated lung injury, suppressed levels of proinflammatory cytokines and oxidative stress in the lungs of the mice under hypoxia. These data suggest that decreased H_2_S level in lung tissue disrupts the balance of pro- and anti-oxidants, enhances oxidative stress and inflammatory responses, and aggravates lung injury. Notably, H_2_S donor treatment could increase SaO_2_ level in the mice with hypoxia insult. Together, it indicates that supplementation of H_2_S might be a potential strategy for improving lung injury and SaO_2_ under hypoxia.

S-sulfhydration has been implicated to be responsible for H_2_S biological function (Ju et al., 2017). Protein sulfhydration of ion channels, transcription factors, and enzymes, has been shown to have protective roles. For instance, H_2_S can inhibit inflammation through sulfhydration of the p65 subunit of nuclear factor-kappa B (NF-κB) at cysteine-38 (Sen et al., 2012). Several studies have demonstrated that H_2_S could sulfhydrate Keap1 at cysteine-151, inducing the dissociation of Nrf2 from Keap1 and enhancing the nuclear translocation of Nrf2. Nrf2 binds to the antioxidant response element (ARE) to promote antioxidant gene transcription (Yang et al., 2013; Guo et al., 2014; Xie et al., 2016). Thus, it is possible that the sulfhydration of NF-κB and Keap1 may be linked to the roles of H_2_S in pulmonary physiological homeostasis. Nevertheless, it requires to be confirmed in the future studies.

In conclusion, our study has demonstrated that CSE deficiency leads to reduced SaO_2_ level and tissue hypoxia in many organs and H_2_S supplementation reverses SaO_2_ level and improves tissue hypoxia. Reduced SaO_2_ level is associated with lung injury but not with airway resistance and blood perfusion in the lungs in CSE deficiency mice. Endogenous H_2_S is involved in the physiological balance between pro- and antioxidants in pulmonary tissues, thereby protecting the lungs against oxidative stress and inflammatory responses. Our findings shed light on the role of H_2_S as a therapeutic agent for hypoxia.

## Data Availability

The raw data supporting the conclusion of this article will be made available by the authors, without undue reservation.
